# Charophytes: Evolutionary Giants and Emerging Model Organisms

**DOI:** 10.3389/fpls.2016.01470

**Published:** 2016-10-10

**Authors:** David S. Domozych, Zoë A. Popper, Iben Sørensen

**Affiliations:** ^1^Department of Biology, Skidmore College, Saratoga SpringsNY, USA; ^2^Botany and Plant Science, School of Natural Science, National University of IrelandGalway, Ireland; ^3^Plant Biology Section, School of Integrative Plant Science, Cornell University, IthacaNY, USA

**Keywords:** charophytes, evolution, model organisms, *Micrasterias*, *Penium*

## Abstract

Charophytes are the group of green algae whose ancestral lineage gave rise to land plants in what resulted in a profoundly transformative event in the natural history of the planet. Extant charophytes exhibit many features that are similar to those found in land plants and their relatively simple phenotypes make them efficacious organisms for the study of many fundamental biological phenomena. Several taxa including *Micrasterias, Penium, Chara*, and *Coleochaete* are valuable model organisms for the study of cell biology, development, physiology and ecology of plants. New and rapidly expanding molecular studies are increasing the use of charophytes that in turn, will dramatically enhance our understanding of the evolution of plants and the adaptations that allowed for survival on land. The *Frontiers in Plant Science* series on “Charophytes” provides an assortment of new research reports and reviews on charophytes and their emerging significance as model plants.

## Introduction

Charophytes or basal Streptophytes; (Becker and Marin, 2009; Leliaert et al., 2012) constitute a diverse taxonomic assortment of extant freshwater and terrestrial green algae that display a wide array of unicellular, filamentous, and “parenchymatous” forms (Graham, 1993; Lewis and McCourt, 2004). An ancestral lineage of charophytes emerged onto and colonized land 450–500 million years ago. These organisms adapted to terrestrial conditions, became capable of surviving and reproducing when fully exposed to the atmosphere, and some members ultimately evolved into land plants (Zhong et al., 2013; Delwiche and Cooper, 2015). This “terrestrialization” of green plants represented a keystone biological event that forever changed the biogeochemistry and natural history of the planet. The subsequent proliferation of land plants changed atmospheric and further altered soil conditions and allowed for the emergence of other diverse life forms onto land. Land plant evolution also transformed human history most significantly through the innovation of agriculture and the consequential creation of modern human civilization. Partly due to their evolutionary significance, charophytes have received significant attention from plant biologists over the past decades (Pickett-Heaps and Marchant, 1972; Pickett-Heaps, 1975; Mattox and Stewart, 1984; Becker and Marin, 2009; Harholt et al., 2016). However, as additional new data has been gathered regarding the biology of these algae, they have become important models for understanding basic phenomena in biochemistry, cell biology, developmental biology, ecology and increasingly, molecular biology (Delwiche and Cooper, 2015; Lemieux et al., 2016). The *Frontiers in Plant Science* series on “Charophytes” illustrates the importance of these organisms in several specific areas of plant biology research. This mini-review highlights the attributes of charophytes as model organisms in diverse areas of research. It also will hopefully provide encouragement for a new generation of scientists to expand the use of these algae in basic research and to initiate screening of other taxa that, in turn, may lead to the identification and use of new model charophytes.

## The “Line-Up” of Charophytes

**FIGURE 1:**
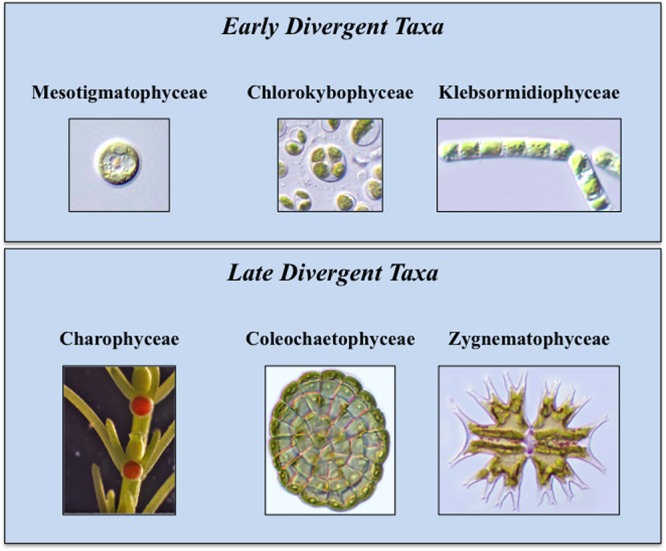
**Extant Charophytes.** Extant Charophytes are divided into early and late diverging taxa. The early diverging taxa include the Mesostigmatophyceae, the Chlorokybophyceae, and the Klebsormidiophyceae and the late diverging taxa include the Charophyceae, the Coleochaetophyceae, and the Zygnematophyceae. Representatives of each group are *Mesostigma* (Mesostigmatophyceae), *Chlorokybus atmophyticus* (Chlorokybophyceae), *Klebsormidium flaccidum* (Klebsormidiophyceae), *Chara* (Charophyceae), *Coleochaete scutata* (Coleochaetophyceae), and *Micrasterias* (Zygnematophyceae).

During the 1970s and 1980s, ultrastructural (e.g., cell division mechanism, flagellar apparatus substructure) and biochemical (e.g., enzyme profiles) data were the main criteria for the inclusion of a green alga in the charophyte lineage (Mattox and Stewart, 1984; Lewis and McCourt, 2004; Leliaert et al., 2012). From the 1990s to today, studies focused on molecular analyses of chloroplast and nuclear genomes and transcriptomes have reaffirmed and refined earlier taxonomic and phylogenetic schemes (Timme et al., 2012; Delwiche and Cooper, 2015). This has further been supplemented by detailed biochemical, immunological and cell biology-based analyses of charophyte cell walls and hormone biosynthetic and signaling pathways (Popper and Fry, 2003; Popper, 2008; Sørensen et al., 2010, 2011, 2012; Zhang and van Duijn, 2014; Ju et al., 2015; O’Rourke et al., 2015). Extant charophytes display a relatively low percentage of diversity in comparison with other green algal taxa and encompass 13 families and 122 genera (Becker and Marin, 2009; Leliaert et al., 2012). Current phylogenetic opinion places the charophytes in six classes (**Figure 1**; Delwiche and Cooper, 2015). The basal class, the Mesostigmatophyceae, is represented by a single known genus, *Mesostigma*. This alga is a unicellular biflagellate with a unique asymmetric shape (i.e., like a flattened lifeboat) and is covered by layers of ornately designed scales (Manton and Ettl, 1965; Becker et al., 1991; Domozych et al., 1991, 1992). The second class, the Chlorokybophyceae, also consists of a single known type, *Chlorokybus atmophyticus.* This rare alga forms a sarcinoid packet of cells surrounded by a thick gel-like covering (Rogers et al., 1980). It should be noted that alternative phylogenies place *Mesostigma* and *Chlorokybus* as sister lineages that together are sister to all other Streptophytes (Rodríguez-Ezpeleta et al., 2007). In the six class phylogeny, the third class, the Klebsormidiophyceae, consists of three genera that form simple unbranched filaments (Sluiman et al., 2008) that often are members of “biological crusts” growing upon surfaces of various terrestrial habitats. Several species in this class can even withstand significant desiccation stress when part of desert crusts (Mikhailyuk et al., 2008, 2014; Holzinger and Karsten, 2013); others are tolerant to the desiccation stresses associated with low temperature environments (Stamenkovic et al., 2014; Herburger and Holzinger, 2015). These first three classes constitute the “early divergent” charophytes. The “late divergent” lineage also includes three classes. The Charophyceae, or stoneworts, are commonly found in freshwater ecosystems and possess macroscopic multicellular thalli consisting of aggregations of branched filaments made of exceptionally elongate cells (Lewis and McCourt, 2004). Members of this group exhibit oogamy-based sexual reproduction that includes motile sperm and non-motile eggs both produced in multicelled gametangia. The Coleochaetophyceae consist of taxa that have multicellular thalli made of branched filamentous or parenchyma-like thalli (Graham, 1993; Delwiche et al., 2002). These organisms also display distinct oogamy-based sexual reproduction. The Coleochaetophyceae are typically found at the interface of freshwater and terrestrial habitats, often as epiphytes. Finally, the largest and most diverse group of charophytes, the Zygnematophyceae, consists of unicells and unbranched filaments (Gontcharov et al., 2003; Guiry, 2013). The distinguishing feature of this group is the presence of conjugation-based sexual reproduction that requires complex cell–cell signaling and adhesion (Abe et al., 2016). The zygnematophycean algae are common inhabitants of freshwater habitats, sometimes occurring in spectacular ephemeral blooms, as well as in moist terrestrial substrates.

## Charophytes as Model Organisms

**FIGURE 2:**
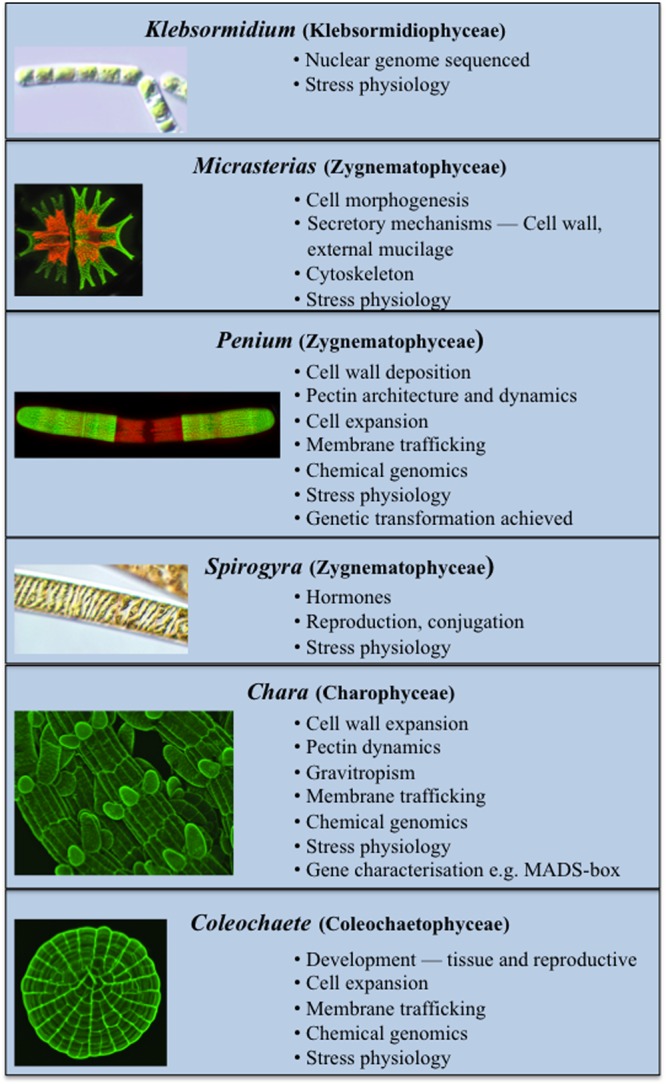
**Current model Charophytes.** Several Charophytes including *Klebsormidium, Micrasterias, Penium, Spirogyra, Chara*, and *Coleochaete* are used as models; each has specific attributes that make it a particularly suitable model for specific physiological and biochemical processes.

Several charophytes have been used extensively as model organisms in the study of basic biological processes. Their small and simple thalli (i.e., when compared to land plants) and ease in experimental manipulation are just two of their attributes that make them attractive model organisms. Recent evidence has also demonstrated that many charophytes have several remarkably similar features to those of land plants including the presence of biosynthetic pathways for many growth regulators (**Figure 2**; Boot et al., 2012; Hori et al., 2014; Wang et al., 2014, 2015; Holzinger and Becker, 2015; Ju et al., 2015) and multiple cell wall polymers (Popper, 2008; Sørensen et al., 2010, 2011, 2012; Mikkelsen et al., 2014). These two characteristics have made charophytes efficacious in such areas of study as plant molecular development and stress physiology. While many charophytes have been used in a wide array of biological studies, the following taxa are most notable for their extensive use in multiple areas of study as models:

### Desmids: The Symmetrical Models

The Zygnematophyceae have recently been shown to most likely be the closest living ancestors of land plants (Wodniok et al., 2011; Delwiche and Cooper, 2015). Unicellular members of the inclusive placoderm desmid group have become important models for elucidating many fundamental principles of plant cell biology and development. Large cell size, notable symmetry/shape, distinct cell wall architecture and elaborate endomembrane/cytoskeletal systems are just a few of the characteristics that make them excellent cell systems for studying cell physiology and development. The recent establishment of stable transformed lines and soon-to-be sequenced genomes/transcriptomes of select desmids have further enhanced their value in botanical studies. Two taxa, *Micrasterias* and *Penium*, have emerged as the most well studied of the desmids.

*Micrasterias* has been the most popular desmid for cell biology research for the past 50 years due to its unique features (Meindl, 1993; Lutz-Meindl, 2016). The *Micrasterias* cell exhibits a bilateral symmetry that is often highlighted by a spectacularly dissected (i.e., multilobed) periphery. New daughter semicells produced by cell division do not have this complex morphology but rather are spherical in shape. A complex post-cytokinetic developmental program that employs multiple sets of highly coordinated subcellular mechanisms subsequently generates the multilobed phenotype during daughter semicell expansion. This program also is responsible for deposition of polymers for the production of both a primary and secondary cell wall. These events are centered on a large network of Golgi bodies and associated vesicles that yield a complex secretory mechanism. This process, in turn, is targeted to specific cell surface sites that generate a multipolar cell expansion and the concurrent secretion of cell wall macromolecules and extracellular mucilage (Kim et al., 1996; Lutz-Meindl and Brosch-Salomon, 2000; Oertel et al., 2004; Aichinger and Lutz-Meindl, 2005). The delivery of secretory components to precise cell surface loci requires an elaborate actinomyosin-based cytomotile system and is controlled by several signal transduction cascades (Meindl et al., 1994; Oertel et al., 2003). Furthermore, cell morphogenesis is highly sensitive to external stress (e.g., oxidative stress, salinity, heavy metals) that leads to major changes in cell differentiation (Darehshouri et al., 2008; Affenzeller et al., 2009; Andosch et al., 2012). *Micrasterias* is an outstanding organism for analyzing these subcellular features as it is easy to maintain and manipulate in the laboratory and it lends itself especially well for acquisition of high resolution imaging using light and electron microscopy including immunocytochemistry, Focused Ion Beam-Scanning Electron Microscopy (FIB-SEM) and Electron Energy Loss Spectroscopy (EELS) imaging (Lutz-Meindl, 2007; Eder and Lutz-Meindl, 2008, 2010; Wanner et al., 2013; Lutz-Meindl et al., 2015). Furthermore, initial molecular analyses including the production of transformed cell lines have further enhanced the use of *Micrasterias* especially in the molecular dynamics of cell wall processing (Vannerum et al., 2010, 2011, 2012).

Over the past decade, the desmid, *Penium margaritaceum*, has also become a valuable model organism (Domozych et al., 2009, 2014; Sørensen et al., 2014; Rydahl et al., 2015; Worden et al., 2015). Unlike *Micrasterias, Penium* has a simple cylindrical shape, possesses only a primary cell wall and deposits wall polymers at two specific loci of the cell surface during expansion (Domozych et al., 2011). This relative simplicity is highly attractive for elucidating fundamental principles of plant cell development including cell wall development, cell expansion and secretion dynamics (Domozych et al., 2005, 2014; Ochs et al., 2014). One of its main attributes is that it has wall polymers similar to those present in many land plants (e.g., cellulose, pectins, hemicelluloses) and that these polymers can be labeled with monoclonal antibodies. After labeling of live cells, these can be returned to culture where subsequent cell expansion and wall deposition events can be monitored (Domozych et al., 2009; Rydahl et al., 2015). *Penium* is also easily maintained in the laboratory and its fast growth rate under precisely controlled conditions makes it an excellent specimen for large-scale concurrent microarray screenings of many chemical agents (by growth in multi-well plates) and for assessment of their specific effects on expansion/wall development (Worden et al., 2015). This significantly aids in revealing the role of specific subcellular components and processes in the expansion/differentiation. Recently, the isolation of stable transformed lines (Sørensen et al., 2014) has further enhanced the potential of this alga in future plant cell studies.

### *Chara* and *Nitella*: Cellular Giants for Plant Physiology

Species of *Chara* and *Nitella* (Charophyceae) have long been used as specimens for a variety of biological investigations especially those dealing with cellular dynamics, expansion and cytoplasmic streaming (Green, 1954; Probine and Preston, 1962). Their macroscopic thalli are distinguished by nodes, where branches and gametangia arise, and internodal regions that consist of exceptionally elongate cells. Internodal cells exhibit a clearly notable stratification of subcellular components whereby helical-oriented chloroplasts define a stationary cortex that surrounds an endoplasm where the fastest recorded actinomyosin-generated cytoplasmic streaming occurs. The cytoplasm contains diverse endomembrane components that participate in dynamic membrane trafficking networks (e.g., endocytosis and exocytosis) that are controlled by complex signal transduction cascades (Foissner and Wastenys, 2000, 2012, 2014; Sommer et al., 2015; Foissner et al., 2016). The internodal cells possess cell walls rich in cellulose and the pectin, homogalacturonan (HG). Turgor is the main driving force for expansion and pectin is most likely the load-bearing component controlling wall expansion. Cyclic, non-enzymatic modulation and concurrent calcium complexing of the HG is the mechanism responsible for controlling wall modulations that lead to cell expansion (Proseus and Boyer, 2007, 2008, 2012; Boyer, 2016). One of the technical advantages of *Chara* and *Nitella* in cellular research is that internodal cells can be individually removed; endoplasm and cell walls can be “dissected out” and then used as acellular systems for experimental manipulation.

Other thallus components like rhizoids have organized polar organization of the cytoplasm. These cells contain sedimentable, mineral-rich statoliths located at the apex that function in gravity perception (Hodick et al., 1998; Braun, 2002; Braun and Limbach, 2006). This feature has made *Chara* the first charophyte in space. During TEXUS rocket parabolic flights, it was shown that the statoliths exert tensional forces on actin filaments. A balance of forces (i.e., gravity and the counteracting force of actin filaments) is responsible for the correct positioning of the statoliths in the rhizoid that, in turn, guarantee the ability to respond to the gravity vector (Braun and Limbach, 2006). The use of laser tweezers and slow rotating centrifuge microscopy has demonstrated that statolith sedimentation is not sufficient to cause gravitropic bending of the rhizoid tip. Rather, the mineral-rich statoliths must settle onto specific regions of the plasma membrane for gravitropic morphological effects to proceed (Braun, 2002). *Chara* has also been used to study other phenomena including electrophysiology and the role and movement of hormones (Belby, 2016).

### *Coleochaete* for Developmental Studies and Pattern Development

The formation of a multicellular thallus (i.e., tissue, organ) of a plant requires precise, spatially regulated cell division planes that are controlled by multiple sets of genes expressed at specific points during development (Besson and Dumais, 2011; Umen, 2014). Directed in both 3-dimensional space and time, thallus morphogenesis also modulates in response to external stress factors. In plants, these developmental programs are made even more complex by the presence of cell walls that do not allow for cell migration or tissue flexibility. The elucidation of the specific events that are central to the manifestation of thallus shape and size is often difficult to decipher in land plants where resolution of specific cell behaviors are often poorly resolved when embedded in highly complex and expansive tissues/organs. The use of organisms with simpler thallus designs has been advantageous for understanding basic developmental phenomena and the charophyte genus *Coleochaete* is one such example. Certain species (e.g., *C. orbicularis, C. scutatum*) produce a parenchymatous discoid thallus that grows outward by a combination of anti- or peri- clinal cell divisions and subsequent expansion of its outermost cells (Brown et al., 1994; Cook, 2004). The plane of division of any cell follows simple rules that are based upon cell size, cell shape and geographic location in the thallus (Dupuy et al., 2010; Besson and Dumais, 2011). This characteristic allows for the construction of mathematical models that can then be used to interpret multicellular morphogenesis (Domozych and Domozych, 2014; Umen, 2014) and yield critical insight into the evolution, biomechanics and physiology of organs and whole organisms. Furthermore, some *Coleochaete* species have been extensively studied to determine the structural and functional modulations that occur to a thallus under desiccation conditions (Graham et al., 2012), i.e., key features in understanding early land plant evolution and plant growth dynamics during droughts.

### Insights from Genomic and Gene Studies

Similarly to many other technologies, molecular tools have the ability to impart new information of importance for many areas of research, including physiology and development. For example members of the MADS-box gene family have been isolated and characterized from three charophytes, *Chara globularis, Coleochaete scutata*, and *Closterium peracerosum-strigosum-littorale* (Tanabe et al., 2005). The expression pattern of the MADS-box genes in the charophytes suggests that they play a role in haploid development and reproduction (Tanabe et al., 2005). They are thought to have been recruited into the diploid generation, and their roles in development further diversified, during land plant evolution (Tanabe et al., 2005). To date *Klebsormidium flaccidum* is the only charophyte for which a near complete nuclear genome is available (Hori et al., 2014); although chloroplast and mitochondrial genomes have been sequenced for representatives of each of the six classes of charophytes and ESTs (expressed sequence tags) are available for some charophytes (Delwiche, 2016). The *Klebsormidium* genome has already revealed the presence of genes for the synthesis of several plant hormones and signaling intermediates, mechanisms for protection against high light intensity (Hori et al., 2014), and the presence of Group IIb WRKY transcription factors that were previously thought to have first appeared in mosses (Rinerson et al., 2015). Several charophyte genome sequencing projects are currently underway (Delwiche, 2016) and will enable more detailed comparative analyses including a larger number of charophytes and potentially give new insight into the evolutionary relationships between species and genes. The unique position of charophytes as the earliest diverging group of Streptophytes makes them a particularly powerful tool for evolutionary-developmental studies and emerging technologies such as the recent achievement of stable transformation in *Penium* (Sørensen et al., 2014) will enable the function of specific genes to be explored.

## Future Uses of Charophytes as Model Organisms

A critical next step in charophyte research is the analysis of the genomes of inclusive taxa and comparative studies with those of land plants. Not only will this work yield key insight into the adaptive mechanisms that charophytes evolved when emerging onto, and successfully colonizing, terrestrial habitats but will further refine and expand the use of current model organisms in fundamental plant research. Although molecular studies will support many areas of research, two areas that will immediately benefit will be cell wall biology and the dynamics of growth regulators in growth and development. In the former, charophytes could provide clarification to many basic yet poorly resolved phenomena including pectin- and cell wall protein- biosynthesis, the controlled secretion, deposition and post-secretory modulations of wall polymers and the specific, interactive membrane trafficking networks in plant cells. In the latter, charophyte models would help decipher the molecular signaling, cell biology and developmental dynamics of growth and development associated with hormones like ethylene and auxin. Recent ecophysiological studies with charophytes also offer a potential bonanza of critical data in determining how plant cells adapt to stress, including desiccation and salt tolerance (Holzinger and Karsten, 2013; Pichrtová et al., 2014; Herburger et al., 2015; Holzinger and Becker, 2015; Holzinger and Pichrtová, 2016; Kondo et al., 2016). The future of charophyte research is indeed very bright and will consequentially become a boon for all of plant biology.

## Author Contributions

All authors listed, have made substantial, direct and intellectual contribution to the work, and approved it for publication.

## Conflict of Interest Statement

The authors declare that the research was conducted in the absence of any commercial or financial relationships that could be construed as a potential conflict of interest.
